# Leaf on a Film: Mesoporous Silica-Based Epoxy Composites with Superhydrophobic Biomimetic Surface Structure as Anti-Corrosion and Anti-Biofilm Coatings

**DOI:** 10.3390/polym16121673

**Published:** 2024-06-12

**Authors:** Jiunn-Jer Hwang, Pei-Yu Chen, Kun-Hao Luo, Yung-Chin Wang, Ting-Ying Lai, Jolleen Natalie I. Balitaan, Shu-Rung Lin, Jui-Ming Yeh

**Affiliations:** 1 Department of Health and Nutrition & Chemical Engineering, Army Academy, Chung Li 320316, Taiwan; jiunnjer1@gmail.com; 2 Center for General Education, Chung Yuan Christian University, Chung Li 320314, Taiwan; 3 Department of Chemistry, Chung Yuan Christian University, Chung Li 320314, Taiwan; 4 Department of Chemistry and Research Center for the Natural and Applied Sciences, University of Santo Tomas, España Boulevard, Manila 1008, Philippines; 5 Department of Bioscience Technology, Chung Yuan Christian University, Chung Li 320314, Taiwan

**Keywords:** superhydrophobic, gas barrier, anti-corrosion, antimicrobial, biomimetic, mesoporous silica, coating

## Abstract

In this study, a series of amine-modified mesoporous silica (AMS)-based epoxy composites with superhydrophobic biomimetic structure surface of *Xanthosoma sagittifolium* leaves (XSLs) were prepared and applied as anti-corrosion and anti-biofilm coatings. Initially, the AMS was synthesized by the base-catalyzed sol–gel reaction of tetraethoxysilane (TEOS) and triethoxysilane (APTES) through a non-surfactant templating route. Subsequently, a series of AMS-based epoxy composites were prepared by performing the ring-opening polymerization of DGEBA with T-403 in the presence of AMS spheres, followed by characterization through FTIR, TEM, and CA. Furthermore, a nano-casting technique with polydimethylsiloxane (PDMS) as the soft template was utilized to transfer the surface pattern of natural XSLs to AMS-based epoxy composites, leading to the formation of AMS-based epoxy composites with biomimetic structure. From a hydrophilic CA of 69°, the surface of non-biomimetic epoxy significantly increased to 152° upon introducing XSL surface structure to the AMS-based epoxy composites. Based on the standard electrochemical anti-corrosion and anti-biofilm measurements, the superhydrophobic BEAMS3 composite was found to exhibit a remarkable anti-corrosion efficiency of ~99% and antimicrobial efficacy of 82% as compared to that of hydrophilic epoxy coatings.

## 1. Introduction

The impact of anti-corrosion and anti-biofilm coatings has extensively attracted a significant number of industrial and research activities in the past decades. Among these coatings, superhydrophobic surfaces usually exhibit remarkable anti-corrosion and anti-biofilm performances. The development of these superhydrophobic surfaces requires distinctive techniques [1], including chemical etching [2], hydrothermal treatment [3], anodization [4], electrodeposition [5], sol–gel processes [6], and templating [7], as well as electrospinning [8]. A study by Yeh et al. replicated the surface structure of a fresh *Xanthosoma sagittifolium* leaf (XSL) to fabricate biomimetic materials containing micron-sized papillae decorated with lots of nano-scaled creases, which exhibited remarkable superhydrophobicity and high surface area [9]. The superhydrophobic property of biomimetic surfaces replicated from XSLs envisioned two applications of corrosion protection [10,11] and anti-biofilm [12]. On the other hand, the high surface area property of biomimetic surfaces envisioned another four distinctive applications of supercapacitors [13,14,15], biological scaffolds [16], photocatalysis [17,18], and gas sensing [19].

Conversely, distinctive hydrophobic metal oxide particles such as SiO_2_, TiO_2_, etc., functioned as inorganic fillers and had been reported to promote the hydrophobicity of organic polymer coatings to give the organic–inorganic hybrid materials. The sol–gel process is a convenient method for preparing different coatings on the surface of galvanized steel substrates to prepare the metal oxide particles. Silica-based protective coatings are very efficient as corrosion protectors of metals under different circumstances [20,21,22]. Mesoporous sol–gel coatings and nanoparticles are suitable as inhibitor carriers for self-healing corrosion-resistant systems [23,24,25]. Recently, novel results and trends were reviewed on this topic thoroughly by Montemor [25]. Silica-based superhydrophobic sol–gel coatings are also promising for preventing metal surfaces against corrosion [26,27,28].

Mesoporous silica with worm-like porosity prepared from a non-surfactant template route was first reported by Wei et al. [29]. Subsequently, Yeh et al. explored the comparative study on the physical properties of PMMA–silica mesocomposite (PSM) and nanocomposite (PSN) membranes [30]. They reported that the PSM was found to show effectively enhanced mechanical strength, thermal stability, thermal insulation, optical clarity, surface hydrophobicity, and surface roughness properties compared to that of the corresponding PSN based on a series of systematic studies. 

Additionally, fillers treated with a silane coupling agent and subjected to ultrasonic treatment can significantly enhance the physicomechanical properties of epoxy composites, improving their impact toughness, heat resistance, and thermal conductivity [31]. After introducing aminopropyl trimethoxysilane as an interfacial modifier to the surface of graphene nanoplatelets, different loadings of silane-modified graphene significantly improved the tensile, compressive, interlaminar shear strength (ILSS), and tribological properties of the epoxy nanocomposites [32]. Hameed et al. investigated the effects of functionalization and different weight fractions of multi-walled carbon nanotubes (CNTs) on the mechanical properties of CNT/epoxy composites. The results showed that increasing the weight fraction of CNTs and applying functionalization treatments significantly enhanced Young’s modulus, tensile strength, and thermal stability of the materials [33].

In this current study, we prepared a superhydrophobic surface of organic–inorganic hybrid materials by integrating the hydrophobic inorganic mesoporous silica particles and hydrophobic biomimetic organic epoxy coating simultaneously for the applications of anti-corrosion and anti-biofilm. Initially, the AMS was synthesized by the base-catalyzed sol–gel reactions of TEOS and APTES through a non-surfactant templating route. Subsequently, a series of AMS-based epoxy composites were prepared by performing the ring-opening polymerization of DGEBA with T-403 in the presence of AMS spheres. Furthermore, a nano-casting technique with PDMS as a soft template was utilized to transfer the surface pattern of natural XSLs to AMS-based epoxy composites, forming AMS-based epoxy composites with biomimetic structure. The anti-corrosion and anti-biofilm of as-prepared materials were intensively characterized by electrochemical corrosion measurements and contact angle measurements.

## 2. Experimental Section

### 2.1. Chemicals and Materials

Tetraethyl orthosilicate (TEOS, Si(OC_2_H_5_)_4_) (reagent grade, 98%), ethanol (C_2_H_5_OH, reagent grade: 99.5%), methanol (CH_3_OH, reagent grade: 99.5%), and glutaraldehyde (C_5_H_8_O_2_, reagent grade: 99%) were all purchased from Acros, Geel, Belgium. D-(-)-Fructose (Fructose, C_6_H_12_O_6_) (reagent grade: 99%), 3-aminopropyltriethoxysilane (APTES, H_2_N(CH_2_)_3_Si(OC_2_H_5_)_3_) (reagent grade, 98.0%), bisphenol A diglycidyl ether (DGEBA, C_15_H_16_O_4_), trimethylolpropane tris[poly(propylene glycol), amine terminated]ether (T-403) were all acquired from Sigma-Aldrich, St. Louis, MO, USA. Ammonium hydroxide solution (NH_4_OH, reagent grade: 25%) was purchased from Fluka, Buchs, Switzerland. Polydimethylsiloxane (PDMS, Sylgard 184 A and B) used for the preparation of the template was purchased from Dow Corning, Midland, MI, USA. N, N-dimethylacetamide (DMAc, C_4_H_9_NO) (reagent grade: 99%) was acquired from Riedel-de Haën, Seelze, Germany.

For antibacterial studies, Luria-–Bertani agar (Cat. No.: LBB500) and nutrient broth (Cat. No.: 213000) were acquired from FocusBio, Taiwan, China, and Becton, Dickinson, and Company, Franklin Lakes, NJ, USA, respectively. *E. coli* (ATCC 23501) and *S. aureus* (ATCC 21351) were all obtained from the Food Industry Research and Development Institute, Bioresource Collection and Research Center, Taiwan, without further modification. All reagents were of reagent grade unless otherwise stated.

### 2.2. Instrumentation

Accelerated Surface Area Porosimetry System (ASAP-2010, Micromeritics Instrument Corporation, Norcross, GA, USA) was used to measure the N_2_ adsorption isotherms of the films. Fourier Transform Infrared Spectrometer (JASCO FT/IR-4100, Easton, PA, USA) was used to gather the IR spectra of samples, employing the Attenuated Total Reflectance (ATR) method. 

Scanning Electron Microscope (SEM, JSM-7600F, JEOL Ltd., Tokyo, Japan), transmission electron microscope (TEM, JEOL 2010, Tokyo, Japan), Fluorescent Microscope (Model: Nikon TE2000-U, Tokyo, Japan), X-ray Diffractometer (XRD, D8 Advance, Bruker, Mannheim, Germany), Static water CA measurements (FACE CBVP-A3, Kyowa Interface Science Co., Ltd., Tokyo, Japan), UV–vis spectrometer (JASCO V-750, Easton, PA, USA) and cyclic voltammetry (CV AutoLab PGSTAT 204, Ω Metrohm Autolab, KM, Utrecht, Netherlands) were used to further characterize the synthesized samples. 

### 2.3. Experimental

This study used D-(-)-Fructose as a pore-forming agent to synthesize mesoporous silica powder. The pore-forming agent was easily removed through washing with water. A direct synthesis approach was employed to modify the surface of silica particles with amine functional groups. The synthesized silica particles were incorporated into an epoxy resin matrix to fabricate organic–inorganic nanocomposites. Subsequent biomimetic techniques were applied to enhance the surface hydrophobicity of the composites. Furthermore, the effects of mesoporous silica and the biomimetic structure on the anti-corrosion properties and anti-biofilm of the nanocomposites were investigated.

#### 2.3.1. Preparation of Amino-Functionalized Mesoporous Silica Spheres (AMS)

DI water (400 mL) and ethanol (800 mL) were combined in a 2000 mL double-walled beaker maintained at 35 °C. Subsequently, 28.8 g of D-(-)-Fructose was added, and the mixture was magnetically stirred until homogeneity was achieved. An NH_4_OH solution (36.0 mL) was slowly introduced into the beaker. TEOS (22.48 g) and APTES (0.5 g) were sequentially added to the mixture using a pipette. Stirring was continued, and after approximately 20 min, the solution transitioned from a transparent state to a milky-white appearance. The mixture was then continuously stirred for 72 h for aging.

**Scheme 1:**
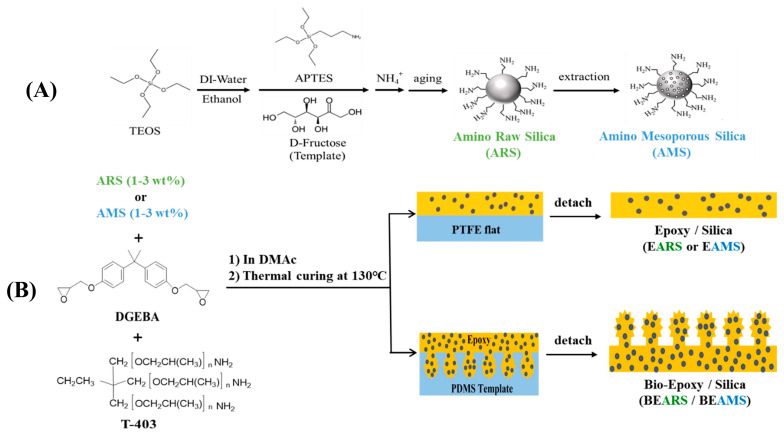
(**A**) Illustration of amino-modified mesoporous silica spheres synthesis. (**B**) Schematic illustration of synthesis of Epoxy/AMS Nanocomposite Coatings with and without an XSL biomimetic structure.

Upon completion of the synthesis, the solution was centrifuged to separate the paste-like material and transferred to a 500 mL beaker. Ethanol (250 mL) was added, and the mixture was subjected to ultrasonic agitation for 30 min. After a subsequent centrifugation for 30 min, the paste-like material was collected. This washing procedure was repeated five times to remove NH_4_OH thoroughly. The obtained material was then placed in a 500 mL beaker, and 250 mL of DI water was added. Ultrasonic agitation was performed for 30 min, followed by centrifugation to retrieve the paste-like substance. This step was repeated ten times to ensure the removal of fructose. After centrifugation, the product underwent lyophilization to yield amino-functionalized mesoporous silica particles. The schematic representation of the synthesis is illustrated in Scheme 1A.

#### 2.3.2. Preparation of *Xanthosoma Sagittifolium Leaves* (XSL)

**Scheme 2:**
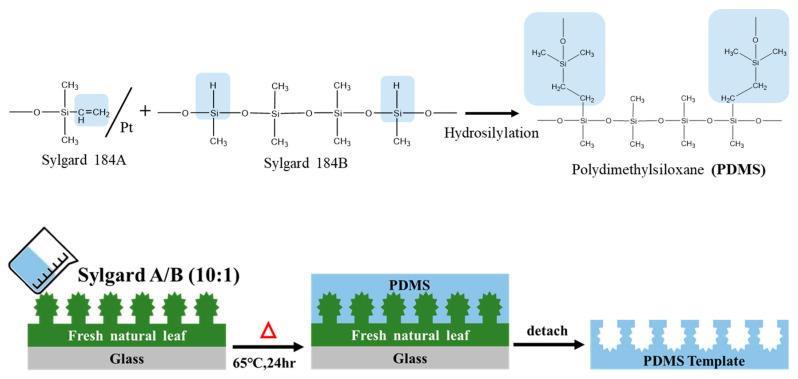
A representation of the preparation process for the PDMS Template with an XSL surface morphology.

Fresh XSLs were thoroughly washed and cut into 2 × 2 cm^2^ sections. These sections were then attached to a glass slide using 3M double-sided tape. A mixture of Sylgard 184A and Sylgard 184B was prepared in a weight ratio of 10:1, and the PDMS solution was stirred until homogeneous. The PDMS mixture was degassed under a vacuum to remove any entrapped air bubbles. The degassed PDMS mixture was subsequently poured over the leaf-affixed glass slide. The slide was then placed in a vacuum oven at 65 °C for 24 h. Once complete crosslinking and solidification occurred, the leaf was carefully peeled off, resulting in a PDMS template replicating the XSL’s surface structure. A schematic representation of this preparation process is illustrated in Scheme 2.

#### 2.3.3. Preparation of Epoxy/AMS Nanocomposite Coatings with and without Biomimetic Structure

An appropriate amount of AMS (Table 1), 1 g of DGEBA, and 1.256 g of DMAc were combined in a 20 mL sample vial. The mixture was subjected to ultrasonic agitation at room temperature and stirred until completely dissolved. Subsequently, 0.424 g of curing agent (T-403) was added to the vial and stirred at room temperature until a uniform phase was obtained. The solution was then spread onto a 5 × 5 cm^2^ PTFE (Teflon) plate and placed on a heating plate with a stepwise heating schedule (50 °C for 1.5 h→80 °C for 1.5 h→110 °C for 1 h→130 °C for 1 h) to complete the polymerization, resulting in a film material without biomimetic structure.

Separately, the solution was spread onto a 2 × 2 cm^2^ PDMS template, and the same stepwise heating procedure was followed. Upon completion of heating and polymerization, the composite film was cooled to room temperature and then carefully peeled off from the PDMS template, yielding an epoxy resin/amino-modified mesoporous silica nanocomposite coating with an XSL biomimetic surface structure. The preparation method is illustrated in Scheme 1B.

### 2.4. Experimental Methods of Electrochemical and Corrosion Properties

Firstly, a Cold-Rolled Steel (CRS) sample was prepared, uniformly coated with a test Epoxy/AMS Nanocomposite with a thickness of approximately 95 µm. After ensuring complete curing of the coating, a conductive silver paste was methodically applied to the uncoated surface of the specimen, subsequently affixing it to a working electrode. This working electrode, in tandem with a saturated calomel electrode (SCE) as the reference electrode and a carbon rod serving as the counter electrode, was immersed in a solution containing 3.5 wt% NaCl. Utilizing the potentiodynamic scanning technique, a potential sweep was executed within a voltage range of −900 mV to −300 mV and a current density range of 1 × 10^3^ to 1 × 10^−5^ µA/cm^2^. The ensuing current was meticulously monitored and recorded. Drawing from these measurements, vital parameters such as the Tafel curve, Corrosion Potential (E*_corr_*), Corrosion Current (I*_corr_*), and Polarization Resistance (R*_p_*) were computed.

For Electrochemical Impedance Spectroscopy (EIS) measurements, we established a current range of 10 nA to 1 A and conducted scans across a frequency spectrum of 0.1 Hz to 1 MHz. The nanocomposites were maintained at a thickness of approximately 95 µm. From these scans, Nyquist and Bode plots were derived. These two graphical representations offer invaluable insights into the protective efficacy of the coating and the interfacial reactions occurring between the material and the corrosive medium. In essence, this experimental methodology furnishes a comprehensive framework for evaluating the anti-corrosive performance of cold-rolled steel, particularly when protected with specific materials.

### 2.5. Antibacterial Adhesion/Growth Experiment

#### 2.5.1. Observation of Bacterial Adhesion via SEM

Membrane samples measuring 1 × 1 cm^2^ were placed into individual wells of 24-well culture plates. A 1.5 mL aliquot of *S. aureus* (ATCC 21351) or *E. coli* (ATCC 23501) bacterial suspension with an OD value of 0.3 was added to each well. The entire setup was transferred to a horizontal shaker incubator with 37 °C and 85 rpm conditions. After 2 and 24 h intervals, the culture plates were removed from the incubator.

Subsequently, the membrane was gently retrieved from the bacterial suspension, rinsed with phosphate-buffered saline (PBS) to remove non-adherent bacteria, and immersed in glutaraldehyde for storing at 4 °C for 24 h. The membrane underwent dehydration the following day via an ethanol gradient series (25%, 50%, 75%, and 100%). The bacterial adhesion on the membrane was then visualized utilizing SEM.

#### 2.5.2. Crystal Violet Staining

EP, EAMS3 composites, and the biomimetic-structured BEP and BEAMS3 membranes were positioned in 24-well culture plates. A 1.5 mL aliquot of *S. aureus* or *E. coli* suspension, with an OD value of 0.3, was introduced to each well. The plates were then transferred to a horizontal shaker incubator at 37 °C and 85 rpm shaking conditions. Post 1 and 7-day incubation, the samples were carefully removed, and any residual, non-adhered bacteria were rinsed away with PBS.

The samples were subsequently stained by immersing them in 1% crystal violet for 5 min. The excess crystal violet was removed by washing the membranes twice using deionized water. These stained samples were mounted onto clean microscopic slides, overlaid with coverslips, and observed under an optical microscope. 

For quantification, 1 mL of methanol was used to solubilize the bound crystal violet from the samples. From this methanol solution, an aliquot of 100 µL was transferred to a 96-well culture plate, and the optical density (OD) was analyzed at 590 nm visual light quantitatively using the microtiter plate reader. Each experimental group was rigorously tested in triplicate to ensure the reliability and reproducibility of the results.

### 2.6. Statistical Analysis

The current study employed a one-way Analysis of Variance (ANOVA) to assess the statistical differences in the antibacterial effects among diverse materials. A *p*-value below 0.05 implies significant differences in the antibacterial efficacies between the materials. 

## 3. Results and Discussion

### 3.1. Characterization and Property Analysis of Mesoporous Materials Prepared via the Non-Surfactant Templating Method

Mesoporous materials are renowned for their high surface area, thereby serving as potential catalyst supports with vast applications in chemical adsorption and catalytic reactions. However, their applicability often becomes constrained when mesoporous materials comprise inert amorphous silica. Enhancing their utility and boosting their stability through surface modification has emerged as a primary research focus in recent studies [2]. This paper explored the concurrent introduction of amino functional groups (amino groups) onto the surface of mesoporous materials using the non-surfactant templating method, followed by a detailed characterization and examination of their morphology and pore structure.

#### 3.1.1. Microscopic Morphology of AMS

**Figure 1:**
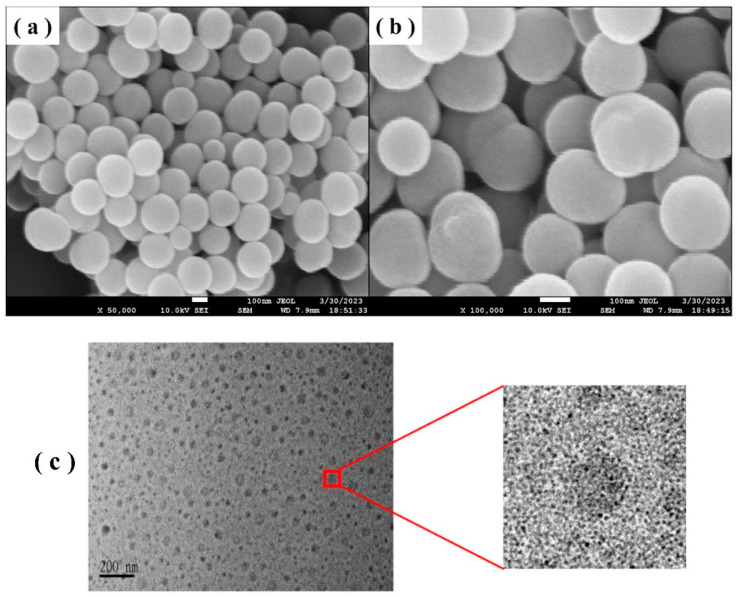
SEM images of aminosilane-functionalized mesoporous silica spheres (AMS) at (**a**) ×50 K magnification and (**b**) ×100 K magnification; (**c**) TEM image of AMS.

Utilizing electron microscopy, the morphology of the synthesized material was observed. SEM images (Figure 1a,b) demonstrate that the average particle diameter of the amino-functionalized silica spheres prepared via the non-surfactant templating method is roughly between 220 and 250 nm post-template removal, with all samples presenting a spherical appearance. The transmission electron microscopy (TEM) images of the mesoporous material of amino-modified AMS produced using the non-surfactant template approach are depicted in Figure 1c. Furthermore, AMS exhibits a notably brighter porous pattern. This suggests that the amino-functionalized mesoporous silica spheres (AMS) are indeed replete with channels, and the distribution of these pores is remarkably uniform.

#### 3.1.2. Chemical Structure Identification and Analysis of Mesoporous Material (AMS)

**Figure 2:**
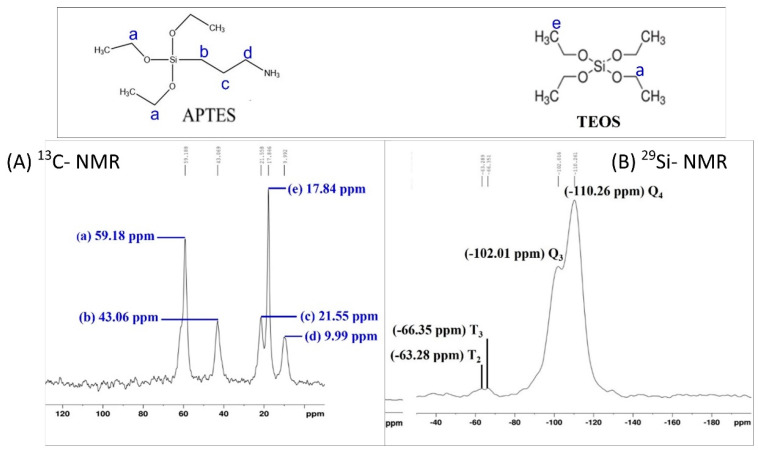
Mesoporous silica spheres (AMS) showing (**A**) ^13^C Solid State NMR spectra and (**B**) ^29^Si Solid State MAS-NMR spectra.

To ascertain whether the mesoporous material successfully incorporated amino functional groups onto the silica surface, we initially utilized the ^13^C-Solid State Nuclear Magnetic Resonance Spectrometer (^13^C-Solid State NMR) for identification. Figure 2A represents the ^13^C-NMR spectrum of the sample derived from the copolymerization of the precursor TEOS and the modifying agent APTES. Carbon positions C(a), C(b), C(c), and C(d) indicated within the APTES structure exhibit chemical shifts of approximately 59 ppm, 43 ppm, 21 ppm, and 10 ppm, respectively. Meanwhile, the carbon positions C(a) and C(e) within the TEOS structure possess chemical shifts of 59 ppm and 17 ppm, respectively. 

When TEOS and APTES undergo hydrolysis followed by sol–gel condensation reactions, most TEOS and APTES form an O-Si-O crosslinked network structure. However, a few silicon atoms would retain either the methyl or methylene functional groups. Moreover, chemical shift signals displayed in the ^29^Si Solid State MAS-NMR spectrum facilitate the determination of the bonding situations between silicon and oxygen atoms within the mesoporous material. Organic silicon (T) and inorganic silicon (Q) chemical structures are predefined in silicon atom structures. In cases where Si-O-Si bonding condensation becomes more complete, electron shielding effects will be reduced, leading to a shift in the absorption peak toward a higher magnetic field. The identification results of the sample’s ^29^Si SS-NMR are depicted in Figure 2B. Upon the sol–gel condensation of TEOS and APTES resulting in AMS, chemical shifts at 102 ppm and 110 ppm are the signals associated with Q^3^ and Q^4^ belonging to the inorganic silicon series. The silica precursor, TEOS, mainly contributes to these. After modification by APTES, signals for T^2^ and T^3^ from the organic silicon series were observed at chemical shifts of −63 ppm and −66 ppm, respectively. By consolidating the evidence from ^13^C SS-NMR and ^29^Si SS-NMR, it can be affirmed that the -NH_2_ active functional group has been successfully bonded within the SiO_2_ mesoporous material [34].

#### 3.1.3. BET Analysis of Mesoporous Properties

**Figure 3:**
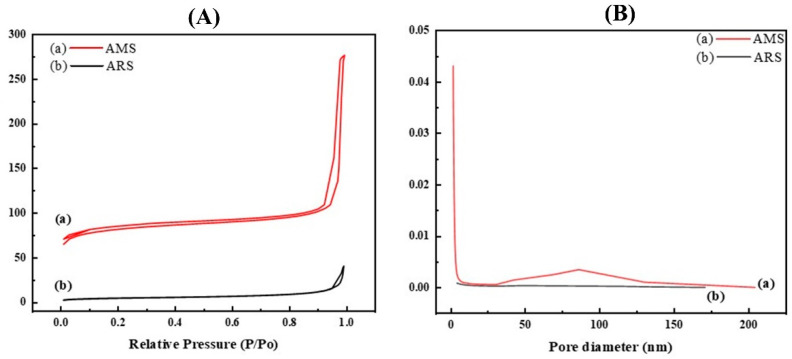
Nitrogen adsorption/desorption isotherms (**A**) and pore size distribution (**B**) for ARS and AMS.

Figure 3A,B represent the nitrogen adsorption/desorption isotherms and pore size distribution of the aminosilane-functionalized silica spheres before (ARS) and after (AMS) template removal, respectively. Before the sample testing, they were heated under a vacuum at 120 °C to remove water vapor and other gases. Subsequently, they were cooled to cryogenic temperatures with liquid nitrogen, followed by nitrogen gas introduction to achieve equilibrium before measurements were taken. From the pore distribution in Figure 3A, the curve is characteristic of a type V isotherm, indicative of a mesoporous material structure. At high P/P_0_, capillary condensation is observed. Using the non-surfactant template D-(-) Fructose, mesoporous materials can be fabricated with an average pore size distribution of approximately 6.4 nm. The specific surface area for ARS is 16.3 m^2^/g, whereas for AMS, it increases to 260 m^2^/g. Likewise, the pore volume for ARS is 0.04 cm^3^/g, and for AMS, it escalates to 0.42 cm^3^/g, marking an approximately tenfold increase. This confirms the successful preparation of mesoporous materials from aminosilane-functionalized silica spheres [34].

### 3.2. The Identification and Application of the Epoxy Resin/Silica Mesoporous Composite with a Structure Biomimetic of the XSF Surface

#### Fourier Transform Infrared Spectra Analysis of Epoxy Resin

**Figure 4:**
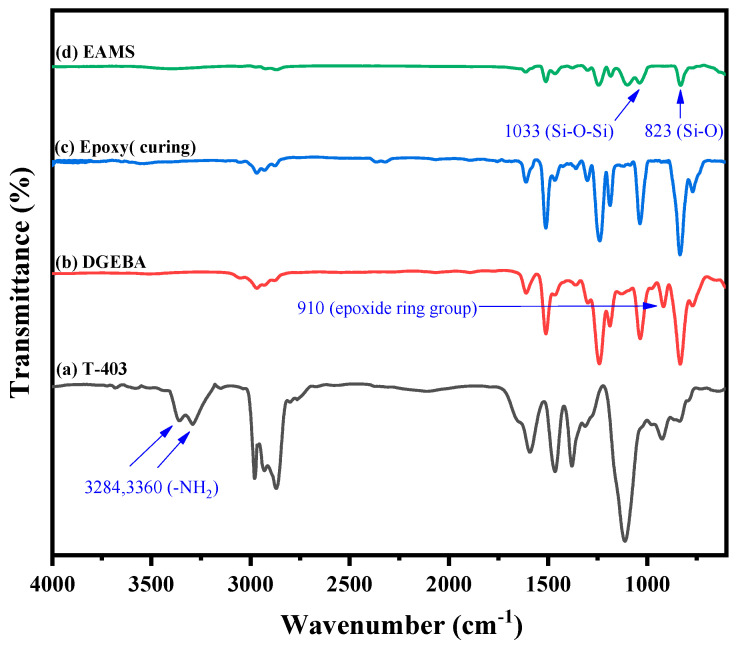
Comparison of FTIR spectra for DEGBA, T-403, and epoxy resin/silica mesoporous composite.

Epoxy resins exhibit high mechanical strength, exceptional electrical insulating properties, and commendable adhesive capabilities. As such, they frequently serve as adhesives, waterproof coatings, IC encapsulants, and functional polymeric materials in fiberglass composites. This study primarily synthesized epoxy resins by uniformly blending the diglycidyl ether of Bisphenol-A (DGEBA) and polyoxypropylene triamine (T-403) in specific ratios. As evidenced by the FTIR spectrum in Figure 4, the hardener with terminal amine groups (T-403) exhibits characteristic peaks of primary amines at absorption positions between 3400 and 3200 cm^−1^. In contrast, DGEBA contains a bis-epoxide resin characterized by epoxy peaks at an absorption position of 910 cm^−1^. As the reaction gradually heated to 150 °C, the ring-opening reaction approached completion. From the epoxy infrared spectrum in Figure 4c, it is apparent that the epoxy absorption peak at 910 cm^−1^ disappeared, as did the characteristic peak of primary amines at 3400–3200 cm^−1^. These changes in the infrared spectra suggest that DGEBA underwent ring-opening, subsequently crosslinking with the primary amine of T-403. Under these reaction conditions, epoxy resins with a high degree of crosslinking can be prepared. Additionally, the epoxy with 3 wt% AMS (EAMS) displays the characteristic absorption peak of Si-O-Si at 1100 cm^−1^ (Figure 4d). This reveals that the mesoporous silica spheres have been successfully mixed into the epoxy resin.

### 3.3. Observation of Surface Morphology of Biomimetic Epoxy/Silica Mesoporous Composites

**Figure 5:**
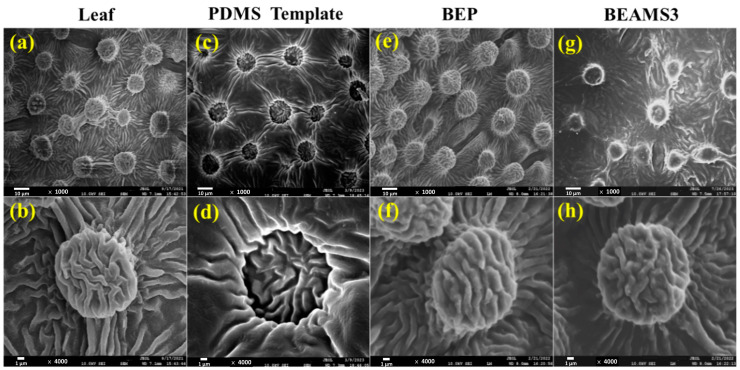
SEM images of surface morphology of biomimetic epoxy/mesoporous silica composite. (**a**,**b**) Natural XSF surface, (**c**,**d**) PDMS template surface, (**e**,**f**) BEP surface structure, and (**g**,**h**) BEAMS3 surface structure.

In this study, Bisphenol A epoxy was employed to transfer the micro-nanostructure of the XSF surface, with subsequent observation and verification of successful replication of the micro-nanostructure using SEM, as presented in Figure 5. Figure 5a,b depict the SEM images of the natural XSF surface morphology. At the same time, Figure 5c,d illustrate the SEM images of the negative mold of the micro-nanostructured XSF leaf back surface, replicated via PDMS imprinting. These images highlight the presence of microscale depressions and nanoscale wrinkles, mirroring the structure found on natural leaves. On the other hand, Figure 5e,f present the SEM images of the surface microstructure of the biomimetic BEP film. The observed microstructure on the BEP coating appears highly reminiscent of the natural XSF morphology, exhibiting numerous microscale papillary structures and nanoscale folding. By incorporating 3 wt% AMS into the epoxy and utilizing the PDMS negative mold for biomimetic imprinting of the XSF surface, the SEM images of the resultant BEAMS3 are depicted in Figure 5g,h. The microstructure on the BEAMS3 coating can be observed, effectively replicating the XSF surface morphology, and featuring microscale papillary formations and nanoscale folding patterns.

**Figure 6:**
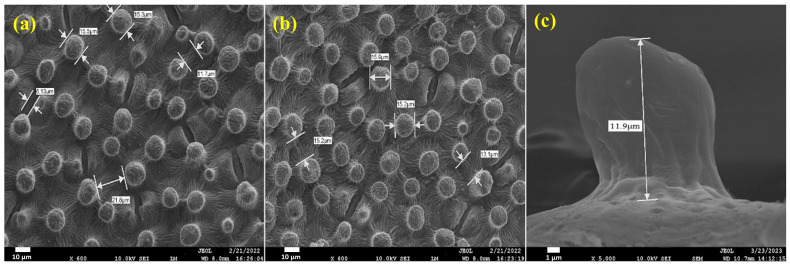
SEM images of horizontal/vertical dimensions of the microstructure on the BEP surface. (**a**) The average distance between the papillae is approximately 13.6 μm, (**b**) the diameter of the papilla is about 15.5 μm, and (**c**) the height of the papilla column is approximately 12 μm.

We subsequently examined the surface morphologies of the biomimetic microstructures on the BEP material, as illustrated in Figure 6. Figure 6a,b show that the average distance between the papillae is approximately 13.6 μm, with a papilla diameter of about 15.5 μm. Additionally, from Figure 6c, the papillary columns’ height is around 12 μm. Through SEM analysis, it is ascertained that the PDMS template can successfully transfer the micro-nano composite structure of the XSF surface. As a result, coatings of BEP and BEAMS3 with micro-nano composite structures are obtained. Further investigations will focus on the influence of these microstructures on the material’s surface properties and related physicochemical analyses.

### 3.4. Static and Dynamic Contact Angle Measurements and Analysis

Using the CA measurement, the hydrophilic and hydrophobic behavior of the epoxy/mesoporous silica composite film was evaluated (Table 2). The contact angle of water droplets on the natural XSF leaf backside is 130°. The non-biomimetic EP coating exhibited a contact angle of 69.2° ± 0.2°, while the biomimetic BEP has an angle of 134.9° ± 0.4°. After introducing the XSF biomimetic template, the originally hydrophilic surface was transformed into a hydrophobic one, increasing the water droplet contact angle by 65°. This validates that the micro-nano structure of XSF indeed enhances the surface hydrophobicity of the material.

**Figure 7:**
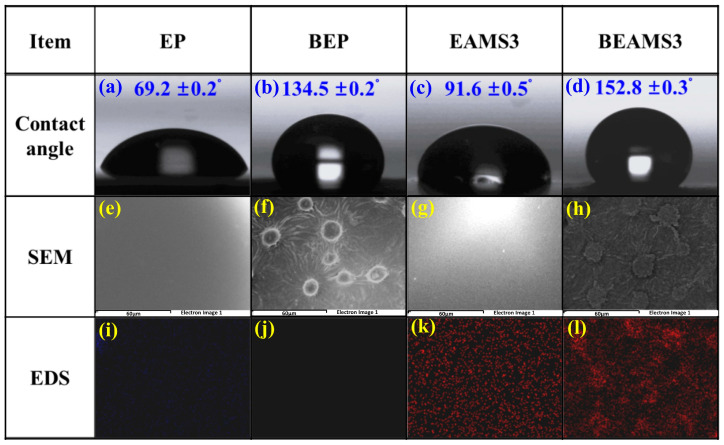
Static contact angle and EDX mapping analysis of silicon elements in the epoxy/mesoporous silica composites.

TEM images from Section 3.1.1 indicate that the AMS is filled with pores internally. The introduction of air blocks water molecules, and the modified silica surface, covered with amino functional groups, enhances the compatibility between inorganic material (AMS) and organic material (epoxy), ensuring a more uniform dispersion of AMS in the epoxy matrix. When AMS is added up to 3 wt%, the static contact angle reaches 91.7° ± 0.5°. Using biomimetic template transfer, by increasing the surface roughness, the contact angle increased to 152.9° ± 0.3°, achieving a superhydrophobic effect (Figure 7). The incorporation of mesoporous silica and biomimetic structure in this study significantly increased the static contact angle by 83°, substantially enhancing the material’s hydrophobicity.

Figure 7i–l present the SEM-EDX mapping analysis for the Si element in the epoxy/mesoporous silica composite. It is evident that when epoxy incorporates AMS, the EDX images display a high distribution of red dots (Figure 7k,l). The uniform distribution of these red dots signifies that the AMS is evenly dispersed within the epoxy.

**Figure 8:**
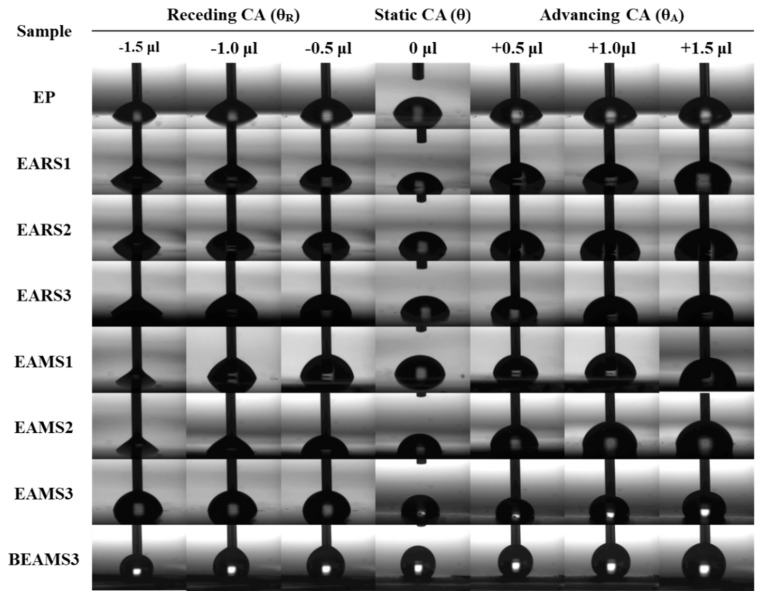
The static water droplet contact angle and dynamic variations of the contact angle induced by the addition and withdrawal of the droplet using a needle for the epoxy/mesoporous silica composites and its biomimetic composites.

Both static and dynamic contact angles play pivotal roles in exploring practical applications for superhydrophobic surfaces. This is attributed to the fact that for materials exhibiting surface roughness or chemical heterogeneities, the contact angle of a droplet does not remain constant but rather fluctuates within a specific range. The maximum and minimum of this range are termed the advancing and receding contact angles, respectively. The difference between these angles is called the contact angle hysteresis (Figure 8.) Typically, the advancing contact angle represents the hydrophobic nature of the surface (corresponding to regions of low surface energy on the solid). In contrast, the receding contact angle reflects its hydrophilic tendencies (corresponding to areas of high surface energy). Notably, a more pronounced roughness on the material’s surface generally increases hysteresis, signifying enhanced hydrophobicity.

This study prepared a series of epoxy/mesoporous silica composites (EAMS1, EAMS2, EAMS3) exhibiting relatively small hysteresis angles ranging from 12° to 22° (Table 2). This suggests a relatively uniform surface, stable surface energy, fewer surface defects, and consistent chemical and physical properties. This further underscores the uniform integration of mesoporous silica with the epoxy matrix. In contrast, biomimetic structures such as BEP and BEAMS3 exhibited a significantly increased hysteresis of 27° and 32°, respectively. Due to the biomimetic XSL surface structure, these materials manifest superhydrophobic properties akin to the lotus leaf effect. Their heightened surface roughness, combined with chemical and physical heterogeneities, alters the interplay between the liquid and solid, leading to distinct advancing and receding angles, thereby resulting in a more significant contact angle hysteresis.

### 3.5. Corrosion Protection Application Testing

#### 3.5.1. Electrochemical Potentiodynamic Testing

Epoxy/mesoporous silica composites were coated onto cold-rolled steel strips to fabricate working electrodes for metal corrosion resistance tests. Parameters such as corrosion potential (E*_corr_*), corrosion current (I*_corr_*), and polarization resistance (R*_p_*) were measured to evaluate the anti-corrosive performance of the coated materials.

The open circuit potential (OCP) was monitored until it reached a stable equilibrium state, after which cyclic potential measurements commenced. Scanning from −500 mV to +500 mV, the resulting data was plotted on a Log I vs. E graph, known as the Tafel plot. This yielded individual anodic and cathodic polarization curves. The intersection of these curves determines the corrosion potential (E*_corr_*), with the corresponding current being the corrosion current (I*_corr_*). Polarization resistance was ascertained by scanning at a rate of 100 mV/s until surpassing the corrosion current. A potential–current graph was plotted, with the resulting slope indicating the polarization resistance (R*_p_*).

In general, a higher corrosion potential (E*_corr_*), a more considerable polarization resistance (R*_p_*), and a smaller corrosion current (I*_corr_*) suggest an enhanced corrosion resistance of the material. The corrosion rate (R*_corr_*) decreases correspondingly. The material’s protective efficiency (P*_EF_*) can be calculated using the following formula:

$$\mathrm{Protection}\;\mathrm{Efficiency}\left(P_{EF}\right)\%=\frac{I_{\mathrm{corr}}\left(CRS\right)-I_{\mathrm{corr}}\left(coated\right)}{I_{\mathrm{corr}}\left(CRS\right)}\times 100\%$$

**Figure 9:**
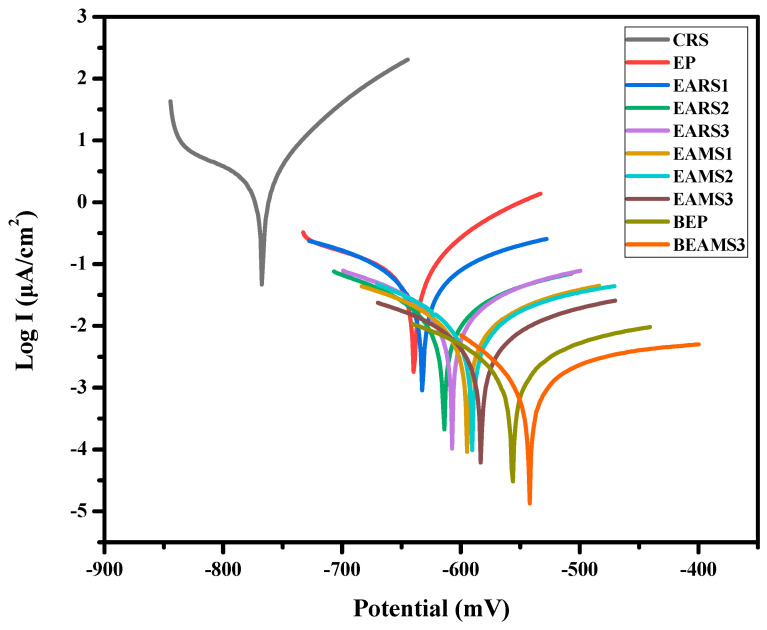
A Tafel plot of the epoxy/mesoporous silica composite material and its biomimetic composite material.

From the Tafel curves shown in Figure 9, one can observe the composite curves formed by both anodic and cathodic polarization curves. The consolidated data are presented in Table 3. A shift of the Tafel curve towards the bottom right indicates an elevated corrosion potential (E*_corr_*) and a decreasing trend in corrosion current. It is hypothesized that including mesoporous silica enhances gas permeation pathways, hindering the diffusion of corrosion agents (water, oxygen), thus achieving corrosion protection. By incorporating 3 wt% AMS into the epoxy resin, its corrosion potential can be improved to −583.9 mV. By further employing a biomimetic surface structure transfer onto this material and conducting corrosion protection tests, its corrosion potential can be further elevated to −556.5 mV. Moreover, its polarization resistance experiences a significant increase, rising from 349 KΩcm^2^ to 15,469 KΩcm^2^. This suggests that the drainage performance inherent to the biomimetic superhydrophobic structure can bolster its anti-corrosion capabilities.

#### 3.5.2. Electrochemical Impedance Testing

Under stable conditions, impedance describes the relationship between current and potential. It is referred to as the Z value when determined using alternating current impedance methods. Electrodes comprise resistance and capacitance, where R_S_ is the electrolyte resistance, R_ct_ is the charge transfer resistance, and C denotes capacitance or the double-layer capacitance. The following equation can express these relationships:

Z = Z′ + j Z″ = R_S_ + R_ct_/(1 + ω j R_ct_ C)

The Nyquist plot derived from this equation is semicircular. A larger radius or area of the semicircle from a low to high frequency indicates higher impedance values, signifying superior barrier properties and corrosion resistance. A more significant real impedance part, Z’, suggests the excellent anti-corrosion performance of the material.

**Figure 10:**
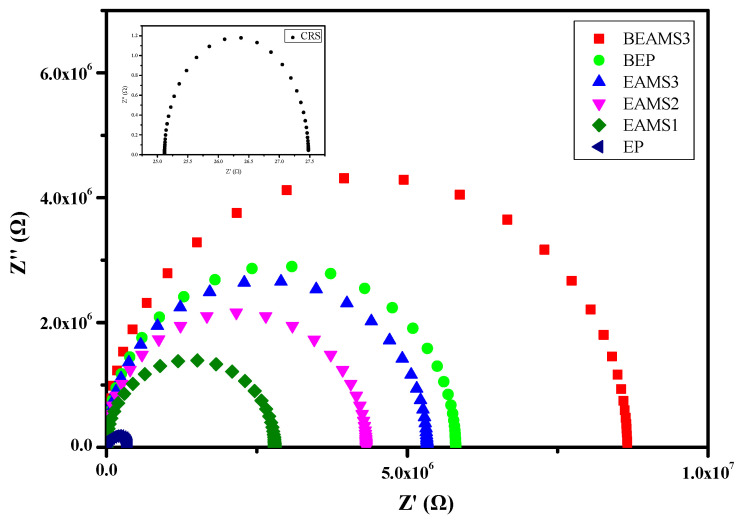
Electrochemical impedance analysis with Nyquist plots for CRS, the epoxy/mesoporous silica composite, and its biomimetic composites.

Figure 10 shows that the epoxy resin/mesoporous silica composite superhydrophobic coating with a biomimetic taro leaf surface structure, labeled BEAMS3, possesses the highest surface impedance. As the content of mesoporous silica gradually increases in the epoxy resin, the impedance value also escalates (Table 3). A primary factor for this is the drainage mechanism of the superhydrophobic surface, which effectively impedes water vapor. Moreover, the uniform dispersion of mesoporous silica within the epoxy resin interferes with oxygen penetration. It prolongs the oxygen pathway, decelerating the onset of rust formation on the metal, thereby substantially enhancing the anti-corrosion effect.

**Figure 11:**
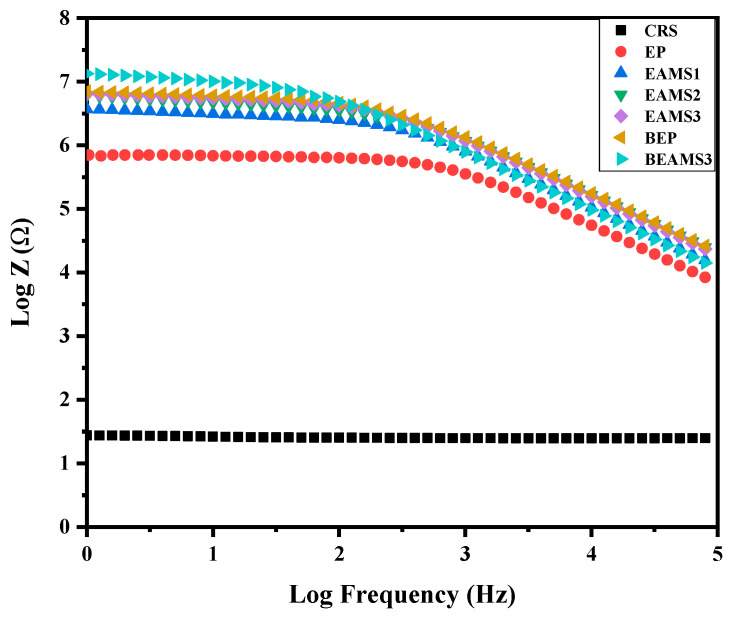
Electrochemical impedance analysis with Bode plots for CRS, the epoxy/mesoporous silica composite, and its biomimetic composites.

In Figure 11, the electrochemical impedance Bode plot reveals trends in the impedance analysis of the epoxy resin/mesoporous silica composite material. These trends align perfectly with the tendencies derived from the Nyquist plot in the electrochemical impedance analysis. Notably, BEAMS3 exhibited the highest impedance value, indicating its superior anti-corrosion effect. Subsequently, the BEAMS3 coating, showcasing the optimal anti-corrosion performance, will be subjected to the next phase of antimicrobial experiments, with EP, BEP, and EAMS3 materials serving as the control group.

### 3.6. Inhibition of Biofilm Formation

**Figure 12:**
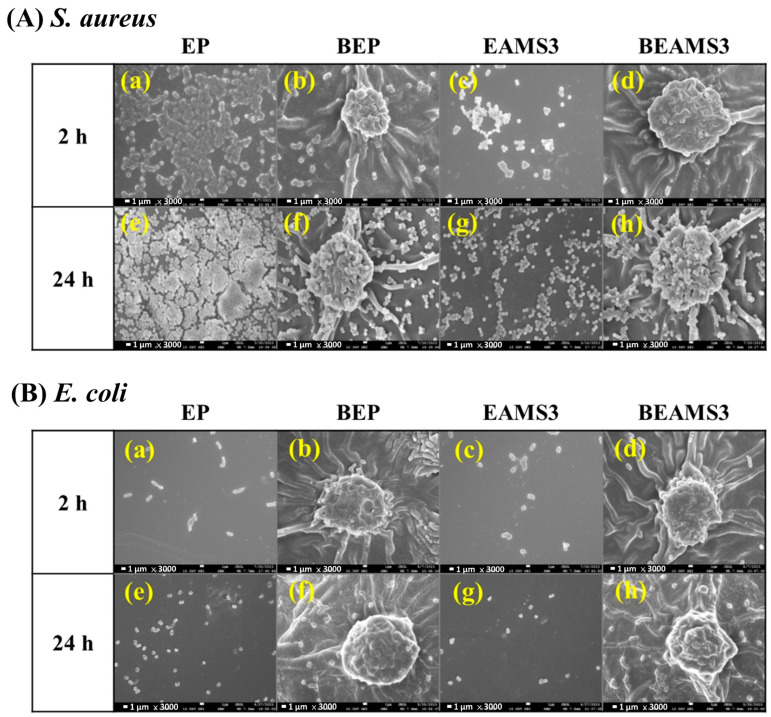
SEM image of bacteria adhesion on films for 2 and 24 h cultivation. (**A**) *S. aureus*; (**B**) *E. coli*. Images are 3000× magnification.

In assessing short-term bacterial attachment inhibition, SEM enabled the observation of bacterial adhesion to the material at 2 and 24 h post-exposure. The images presented in Figure 12 depict the material’s surface morphology amplified 3000-fold under SEM.

The findings of *S. aureus* attachment to the materials after 2 and 24 h incubation are shown in Figure 12, in which a large number of clustered cocci on the EP are spread all throughout the surface of EP (Figure 12(Aa,e)). However, only a few sporadic cocci appeared on the surface of EAMS3, BEP, and BEAMS3 (Figure 12(Ab,f)). After soaking the materials for 24 h, a cocci-covered EP surface was found (Figure 12(Ae)). Meanwhile, only a few cocci grew within the space between the papillae in EAMS3, BEP, and BEAMS3 (Figure 12(Ab–d,f–h)), possibly due to the limited attachment and proliferation of cocci to the papillae. 

Figure 12B illustrates the adherence of *E. coli* to the materials following 2 and 24 h of incubation. Notably, only a few *E. coli* cells were observed adhering to both EP and EAMS3 surfaces with the bacteria uniformly distributed across these surfaces. On the BEP and BEAMS3 films, *E. coli* attachment was markedly less frequent, with only isolated bacteria noted after a 2 h incubation (Figure 12(Ba,c)). The panels from Figure 12(Be–h) detail the extent of bacterial colonization on the materials after a prolonged 24 h soaking period. The EP and EAMS3 surfaces were covered with *E. coli*. Contrastingly, *E. coli* was only sporadically observed on the BEP and BEAMS3 surfaces. These materials exhibited superior efficacy in inhibiting bacterial attachment compared to EP and EAMS3. The aforementioned findings underscore that the biomimetic surfaces of BEP and BEAMS3 effectively restrict the proliferation of both bacterial strains.

**Figure 13:**
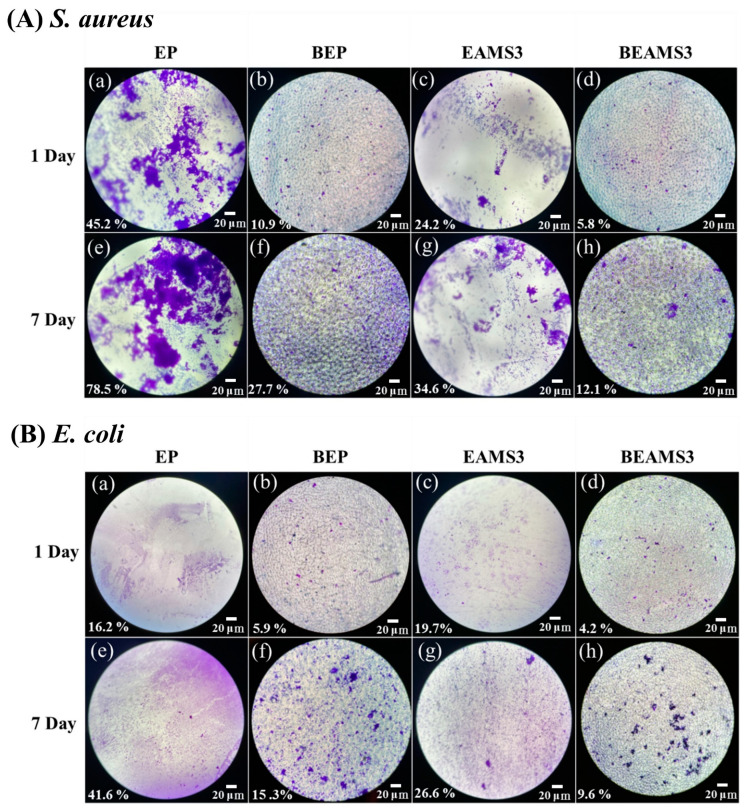
Image of crystal violet staining of bacteria growth on films for one day and seven days. (**A**) *S. aureus*; (**B**) *E. coli*. Coverage is also indicated at bottom-left corner.

In evaluating the long-term anti-biofilm efficacy of the materials, crystal violet (CV) staining was employed for quantitative analysis, complemented by a microscopic examination to visualize biofilm development. The CV staining distinctly marked the bacterial presence, as indicated by the purple regions on the material. In this study, a biofilm inhibition assay was performed using *S. aureus* and *E. coli* over periods of 1 and 7 days. Figure 13A depicts the biofilm formation of *S. aureus* on the material at these time intervals. After the initial day of bacterial exposure, a more pronounced white area was noticeable on the EP, suggesting a lower extent of biofilm formation with limited bacterial adhesion (45.2% coverage) (Figure 13(Aa)). By the 7th day, the white area had noticeably diminished, indicating a substantial increase in biofilm presence (Figure 13(Ae)). The purple spots distributed across the image reflect a significant escalation in bacterial accumulation on the material (78.5% coverage). For EAMS3, a smaller number of purple spots were formed throughout the image, indicating a remarkable decrease in bacteria on the material with a reduced coverage of 24.2% and 34.6% for days 1 and 7 (Figure 13(Ac,g)). It indicates that EAMS3 has better anti-biofilm properties than EP. For BEP and BEAMS3 with a superhydrophobic surface, there are only a few purple spots throughout the image indicating a remarkable decrease in bacteria on the material with a reduced coverage of 10.9% and 5.8% for day 1 (Figure 13(Ab,d)) and 27.7% and 12.1% for day 7 (Figure 13(Af,h)), respectively. It indicates that BEP and BEAMS3 have better anti-biofilm properties for their biomimetic surface structure.

The proliferation of *E. coli* on the material was also monitored and compared over 1 and 7 days (Figure 13B). Initially, after 1 day of bacterial growth, scattered purple spots indicating limited bacterial presence (16.2% coverage) were observed on the EP, suggesting that biofilm formation was still in its early stages (Figure 13(Ba)). By contrast, on day 7, the density of purple spots markedly increased on the material, denoting a substantial escalation in *E. coli* growth (41.6% coverage), indicative of significant biofilm development (Figure 13(Be)). For EAMS3, a smaller number of purple spots were formed throughout the image, demonstrating a remarkable decrease in bacteria on the material with a reduced coverage of 19.7% and 26.6% for days 1 and 7 (Figure 13(Bc,g)). This indicates that EAMS3 has better anti-biofilm properties than EP. However, the superhydrophobic BEP significantly inhibited the growth of *E. coli* with a reduced coverage of 5.9% and 15.3% for day 1 and day 7 (Figure 13(Bb,f)). These findings indicate that BEP’s surface is relatively inhospitable to *E. coli*, with only minimal bacterial growth observed. This suggests that BEP possesses enhanced anti-biofilm capabilities compared to EP. Furthermore, the superhydrophobic BEAMS3 exhibited almost complete inhibition of *E. coli* proliferation, with bacterial coverage limited to just 4.2% and 9.6% on day 1 and day 7, respectively (Figure 13(Bd,h)). This evidence strongly suggests that the growth of *E. coli* on BEAMS3 is significantly impeded, underscoring its exceptional anti-biofilm properties.

**Figure 14:**
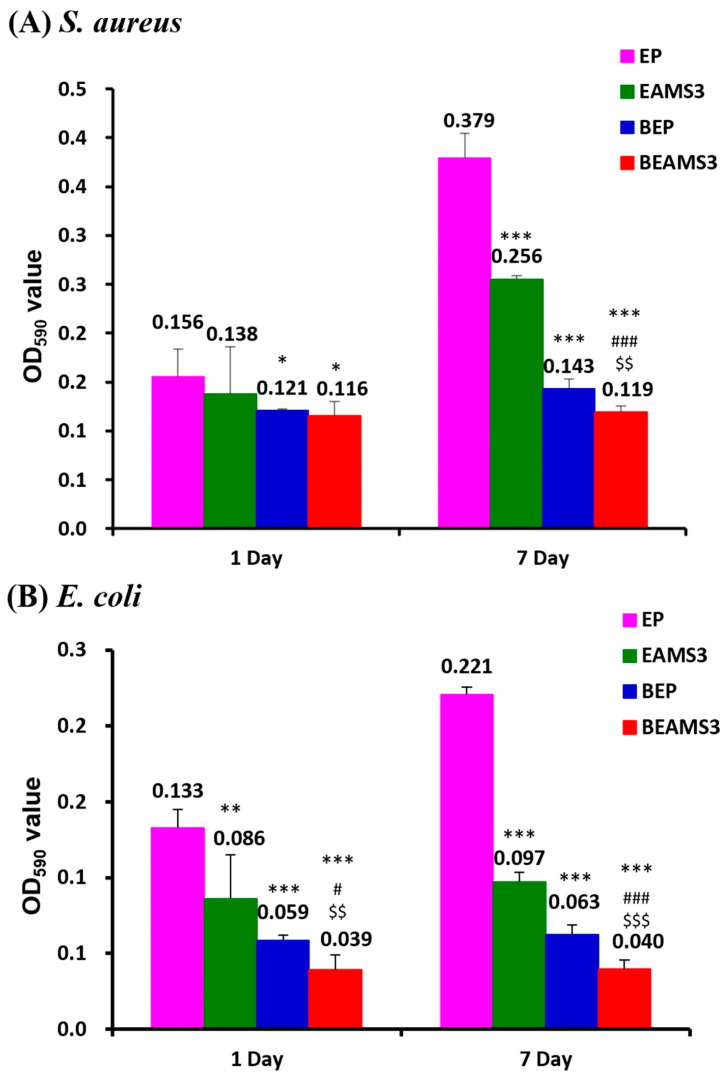
Anti-biofilm performance analysis of EP, EAMS3, BEP, and BEAMS3 nanocomposites membranes against *S. aureus* and *E. coli*. EP, EAMS3, BEP, and BEAMS3 were tested against two bacteria after 1 and 7 days of incubation for anti-biofilm properties. The solubilized crystal violet was quantified by OD_590_ and shown as mean ± SD. (**A**) *S. aureus*; (**B**) *E. coli*. An ANOVA test was conducted for statistical analysis. *: films compared with EP; #: BEAMS3 compared with EAMS3. *, #: *p* < 0.05; **, $$: *p* < 0.005; ***, ###, $$$: *p* < 0.0005.

OD_590_ values showed the quantitative CV stain data in Figure 14. For *S. aureus*, significant differences (*p*-value < 0.05) were observed in BEP and BEAMS3 membranes on day 1, and EAMS3, BEP, and BEAMS3 membranes on day 7, when compared with the EP membrane. In addition, significant differences were also observed in BEAMS3 when compared with EAMS3 or BEP, which proved that BEAMS3 has the best anti-biofilm formation property (Figure 14A). For *E. coli*, the OD_590_ values significantly decreased in EAMS3, BEP, and BEAMS3 membranes both on day 1 and day 7 when compared with the EP membrane. Moreover, significant decreases were also observed in BEAMS3 compared to EAMS3 or BEP on day 1 and day 7 (Figure 14B). All these results proved that BEAMS3 has the best anti-biofilm formation property.

The reduction in bacterial adhesion/growth was calculated using the following formula for each AMS3-contained or micro-structured EP membrane:

(1) $$\mathrm{Efficiency}\;\mathrm{of}\;\mathrm{biofilm}\;\mathrm{inhibition}\;(\%)=\frac{\left(\mathrm{OD590}\;\mathrm{value}\;\mathrm{of}\;\mathrm{EP}\;\mathrm{membrane}\right)-\left(\mathrm{OD590}\;\mathrm{value}\;\mathrm{of}\;\mathrm{Sample}\;\mathrm{membrane}\right)}{\left(\mathrm{OD590}\;\mathrm{value}\;\mathrm{of}\;\mathrm{EP}\;\mathrm{membrane}\right)}\times 100\%$$

Sample membranes include EAMS3, BEP, or BEAMS3.

Table 4 shows that after a day of *S. aureus* cultivation, the efficiencies of biofilm inhibition in EAMS3, BEP, and BEAMS3 were 11.2%, 22.2%, and 25.8%, respectively. On day 7, the number of attached/grown bacteria did not continue to increase with higher antibacterial properties, 32.6%, 62.3%, and 68.6%, respectively.

For *E. coli*, the bacterial growth in the membrane had a similar trend and better results compared to that of *S. aureus* (Table 4). The efficiencies of biofilm inhibition in EAMS3, BEP, and BEAMS3 were 35.1%, 56.0%, and 70.5%, respectively, after one day of cultivation. With the increase in time (day 7), the antibacterial properties increased to 55.9%, 71.7%, and 82.0%, respectively. These results prove that adding AMS3 and biomimetic feather micro-structures could specifically inhibit biofilm formation.

## 4. Conclusions

This study successfully prepared a series of amine-modified mesoporous silica (AMS)-based epoxy composites with superhydrophobic biomimetic structures mimicking the surface of *Xanthosoma sagittifolium* leaves (XSLs). By integrating the concepts of “gas barrier properties” and “hydrophobicity”, the materials were endowed with dual-functional properties of anti-corrosion and antibacterial. A biomimetic negative mold of PDMS replicating the micro-nanostructure of XSL surfaces was prepared through soft lithography, synthesizing BEMS3 materials with biomimetic structures and adding 3 wt% AMS. The contact angle of water droplets on the material increased from 69° to 152°, significantly improving by 83°, and exhibiting superhydrophobic performance. Electrochemical corrosion tests demonstrated that the addition of inorganic filler (AMS) could increase the gas permeation path, obstructing the diffusion of corrosive factors (water, oxygen) and further enhancing anti-corrosion capability, with the polarization resistance (Rp) increasing from 349 KΩcm^2^ to 20,292 KΩcm^2^, achieving a protection efficiency of up to 99.95%.

In terms of antibacterial applications, short-term bacterial anti-adhesion effects were observed with SEM, revealing that the BEMS3 material, with its biomimetic structure and the addition of 3 wt% AMS, exhibited the best anti-adhesion effect against both *S. aureus* and *E. coli*. The long-term anti-biofilm growth effect was quantitatively monitored using microscopy and an Elsa Reader. After a 7-day test period against *E. coli*, the BEMS3 composite material achieved an antibacterial efficiency of up to 82%, maintaining the best long-term anti-biofilm growth effect. In both antibacterial tests, the effect was better against *E. coli*, as the shape and size of bacteria could affect their adhesion to the membrane, with *S. aureus* being closer in size to the gaps on the BEAMS3, making it easier to adhere compared to rod-shaped bacteria [12]. The geometric shape, size, and height of the surface biomimetic structures influenced the contact area and the number of bacteria adhering to the membrane surface, limiting the motility of flagellated *E. coli* and delaying direct contact between bacteria, thus inhibiting biofilm formation. The papillary nanostructures of XSLs and the air layer formed an antibacterial mechanism on the superhydrophobic surface to reduce the bacterial adhesion area [13].

The presence of air, moisture, and salts in the atmospheric environment promotes the corrosion of metal materials and provides suitable conditions for bacterial growth. Based on this, the study developed a dual-functional coating with anti-corrosion and antibacterial properties, addressing the need for metal corrosion prevention and antibacterial action in daily life and preventing biofilm formation. It is also hoped that this coating can be used in food processing facilities to inhibit microbial growth and prevent the corrosion of processing equipment, thereby avoiding food contamination. Moreover, the coating can be applied to marine environments to resist microbial and seawater corrosion, thus reducing damage to ships, offshore platforms, and other metal structures, and ensuring the safety of personnel and property. The development of this innovative coating not only addresses the dual challenges of metal corrosion and microbial growth but also opens new possibilities for future application areas.

## Figures and Tables

**Table 1:** Sample designation and formulation ratios for Epoxy/AMS Nanocomposite Coatings.

Sample Code	Full Name of Sample	DGEBA (g)	T-403 (g)	DMAc (g)	Silica	Silica Loading(g)	AMS (wt%)
EP	Epoxy resin	1	0.424	1.256	--	0	0
EAMS1	Epoxy/amino-modified mesoporous silica nanocomposite	1	0.424	1.256	AMS	0.05	1
EAMS2		1	0.424	1.256	AMS	0.10	2
EAMS3		1	0.424	1.256	AMS	0.15	3
BEP	Biomimetic Epoxy	1	0.424	1.256	--	0	0
BEAMS3	Biomimetic Epoxy/amino-modified mesoporous silica nanocomposite	1	0.424	1.256	AMS	0.15	3

**Table 2:** Data on the static and dynamic water droplet contact angles, as well as the hysteresis angle, for the epoxy/mesoporous silica composites.

Sample Code	Receding Angle (θ_R_)	Static Contact Angle (θ)	Advancing Angles (θ_A_)	Hysteresis (H)
XSF Leaf		130.87° ± 0.2		
EP	66.90°	69.27° ± 0.2	71.53°	4.63°
EAMS1	70.75°	78.90° ± 0.1	83.67°	12.92°
EAMS2	67.94°	81.18° ± 2.5	83.31°	15.36°
EAMS3	82.56°	91.67° ± 0.5	104.99°	22.43°
BEP	115.33°	134.85° ± 0.4	14.42°	27.09°
BEAMS3	123.24°	152.86° ± 0.3	154.46°	31.22°

**Table 3:** Corrosion data from Tafel tests, including the corrosion potential (E*_corr_*), corrosion current (I*_corr_*), polarization resistance (R*_p_*), and protection efficiency (P*_EF_*), as well as impedance tests with Bode plots for CRS, the epoxy/mesoporous silica composites, and its biomimetic composites.

Sample Code	Electrochemical Corrosion Measurements				P*_EF_* (%)	Electrochemical Impedance		Thickness (μm)
	E*_corr_* (mV)	R*_p_* (KΩcm^2^)	I*_corr_* (μA/cm^2^)	R*_corr_* (m/Year)		Z’ (Ω)	log Z (Ω)	
CRS	−767.21	82.8	0.3256	3.784	-	27.48	1.41	-
EP	−639.39	349	0.0092	0.107	97.17%	3.67 × 10^5^	5.38	92 ± 1.4
EAMS1	−594.57	3185	0.0009	0.011	99.72%	2.79 × 10^6^	5.77	92 ± 0.7
EAMS2	−590.56	3295	0.0007	0.009	99.77%	4.32 × 10^6^	5.92	94 ± 2.1
EAMS3	−583.09	6316	0.0005	0.006	99.84%	5.33 × 10^6^	5.97	93 ± 1.4
BEP	−556.39	14,655	0.0003	0.004	99.90%	5.81 × 10^6^	5.98	95 ± 1.3
BEAMS3	−542.19	20,292	0.0001	0.002	99.95%	8.66 × 10^6^	6.00	97 ± 2.3

**Table 4:** Antibacterial efficiency of EAMS3, BEP, and BEAMS3.

	*S. aureus*		*E. coli*	
Sample	1 Day	7 Day	1 Day	7 Day
EP	-	-	-	-
EAMS3	11.2%	32.6%	35.1%	55.9%
BEP	22.2%	62.3%	56.0%	71.7%
BEAMS3	25.8%	68.6%	70.5%	82.0%

## Data Availability

Data are contained within the article.
